# Development and Validation of a Risk Assessment Model for Venous Thromboembolism in Patients With Invasive Mechanical Ventilation

**DOI:** 10.7759/cureus.27164

**Published:** 2022-07-23

**Authors:** Jiajia Lin, Yue Zhang, Weixian Lin, Ying Meng

**Affiliations:** 1 Departments of Respiratory and Critical Care Medicine, Nanfang Hospital, Southern Medical University, Guangzhou, CHN

**Keywords:** critically ill, risk assessment model, invasive mechanical ventilation, intensive care unit, venous thromboembolism

## Abstract

Background

Patients with invasive mechanical ventilation may be at high risk of acquiring venous thromboembolism (VTE). We aim to develop risk assessment models for predicting the improvement of VTE in invasively ventilated patients.

Methodology

A total of 6,734 invasively ventilated patients enrolled from the Medical Information Mart for Intensive Care-III (MIMIC-III) database were used as input for model development and internal validation, while data from 168 patients from Nanfang Hospital were used for external validation. Logistic regression was performed based on predictive factors derived from least absolute shrinkage and selection operator (LASSO) regression analysis and logistic regression with backward selection to develop two Risk Assessment Models (RAM), namely, I and II, for the prediction of VTE, respectively. Model selection was performed by evaluation of the area under the receiver operating characteristic curve (AUC), the goodness of fit with calibration curves, and decision curve analyses (DCA).

Results

RAM-I included prior history of VTE, in-hospital immobilization, infection, glucose, the use of antiplatelet, and activated partial thromboplastin time (APTT) as variables, while RAM-II included prior history of VTE, in-hospital immobilization, infection, ischemic stroke, glucose, the use of antiplatelet and APTT as variables. Compared with RAM-I and ICU-Venous Thromboembolism Score, RAM-II exhibited better discrimination in the training dataset (AUC = 0.826), internal validation dataset (AUC = 0.771), and external validation dataset (AUC = 0.770). Additionally, DCA demonstrated that RAM-II was clinically beneficial. Inspection of the calibration curves revealed good agreement between the predictions and observations.

Conclusions

A RAM for VTE in invasively ventilated patients was developed with reasonable performance.

## Introduction

Venous thromboembolism (VTE) is the third leading cause of cardiovascular-related death occurrences worldwide after acute myocardial infarction and stroke [1]. VTE commonly presents as deep vein thrombosis (DVT) of the lower extremities and pulmonary embolism (PE). Patients in the intensive care unit (ICU) may be at high risk of presenting with VTE due to underlying medical illnesses, prolonged immobilization, and the need for mechanical ventilation [2]. The incidence of VTE in critically ill patients ranges between 7.5% and 31% [3], which is almost twice as high as patients in general wards [2]. On the other hand, patients presenting with VTE complications tend to have longer ICU and hospital stays, as well as longer duration of mechanical ventilation, compared to those without VTE [4].

Prophylactic anticoagulation is recommended for all critically ill patients without contraindications [5]. However, many patients still develop VTE, even if appropriate prophylactic anticoagulation has been enforced, especially those requiring prolonged mechanical ventilation [2]. Undetected PE may prolong the stay from mechanical ventilation. Using therapeutic-dose anticoagulants directly without imaging proof can increase the risk of bleeding among invasively ventilated patients. Repeated and goal-directed bedside ultrasonography allows physicians to monitor the condition of the veins of the patient’s extremities. Nevertheless, VTE remains one of the most common unsuspected autopsy findings in critically ill patients [6]. Moreover, the necessity of computed tomography pulmonary angiography (CTPA) should be carefully evaluated due to the risk during transportation and nephrotoxicity caused by contrast agents. Therefore, risk assessment of invasively ventilated patients for more aggressive prophylactic strategies should be considered to reduce the potential morbidity associated with untreated VTE in this high-risk population [2].

Although scores for the risk prediction of VTE among hospitalized patients exist, a study has shown that Wells and revised Geneva scores are not reliable predictors of PE for patients in the ICU [7]. Compared to non-critically ill patients, invasively ventilated patients are under prolonged immobilization, have complicated comorbidities, and undergo several invasive treatments, which are variables that were not included in most risk prediction models. Recently, a VTE risk prediction tool for critically ill patients, namely, ICU-Venous Thromboembolism Score, has been developed [8]. However, there is no research focusing on external validation of this score, especially among invasively ventilated patients. In this study, two new predictive models were developed in different ways for the occurrence of VTE in invasively ventilated patients, and the performance of the new models was compared with the ICU-Venous Thromboembolism Score to determine the best model.

## Materials and methods

Data source

The relevant data from 6,734 patients were retrieved from the Medical Information Mart for Intensive Care-III database version 1.4 (MIMIC-III v1.4) and was used for model development and internal validation. Data from a total of 168 patients from Nanfang Hospital were used for external validation. The MIMIC-III database is a free-access database comprising health-related data from more than 40,000 patients who stayed in the ICU of the Beth Israel Deaconess Medical Center between 2001 and 2012. The use of the MIMIC-III database was under the approval from the review boards of the Massachusetts Institute of Technology and Beth Israel Deaconess Medical Center. To protect patient privacy, patient data in the database are anonymous; hence, the requirement for informed consent was waived. This study was conducted in accordance with the recommendations of the Transparent Reporting of a multivariable prediction model for Individual Prognosis Or Diagnosis (TRIPOD) statement.

Definition of venous thromboembolism

VTE was defined as acute incident DVT (either upper or lower extremity), superficial vein thrombosis (either upper or lower extremity), or PE that was diagnosed using the International Classification of Diseases and Ninth Revision (ICD-9) codes in the MIMIC-III database or according to discharge diagnoses in Nanfang Hospital. The reports of duplex venous ultrasonography, CT venography, or CTPA were manually reviewed to confirm the diagnosis of VTE. The diagnosis date of VTE was defined as the date of imaging confirmation.

Study participants

Adult patients (aged ≥18 years) who required invasive mechanical ventilation were included in this study. The first admission of a patient was analyzed in case multiple admissions existed. Patients were excluded meeting some of the following criteria: (1) patients who had VTE before or within 24 hours after invasive mechanical ventilation; (2) patients who were diagnosed with chronic venous embolism or chronic pulmonary embolism using ICD-9 codes; and (3) patients with more than four missing baseline variables. Patients from the MIMIC-III database were randomly divided to form the training dataset and internal validation dataset at a ratio of 7:3. The training dataset was used to establish risk assessment models, while the internal validation dataset and external validation dataset were used for validation.

Data extraction

We extracted data from the MIMIC-III database using structure query language (SQL) with PostgreSQL (version 12.4.1, www.postgresql.org) and Navicat Premium (version 15.0.18, www.navicat.com.cn). Clinical electronic medical records were reviewed, and data were manually collected from Nanfang Hospital. The variables in this study included demographic information, symptoms, vital signs, comorbidities, laboratory parameters and treatments. Demographic data included age, gender, and duration of in-hospital immobilization. Symptoms included edema, pain in the extremities, and chest pain. Vital signs included maximum systolic blood pressure (SBP), maximum diastolic blood pressure (DBP), maximum heart rate (HR), maximum respiratory rate (RR), and minimum SpO_2_, all of which were collected within 24 hours before or after invasive mechanical ventilation. Comorbidity data from the MIMIC-III database were collected for analysis based on ICD-9 codes, while data from Nanfang Hospital were based on discharge diagnoses, including prior history of VTE, congestive heart failure, chronic pulmonary disease, diabetes mellitus, hypertension, liver disease, malignant tumor, infection, acute myocardial infarction, atrial fibrillation or atrial flutter, stroke, and trauma. Laboratory variables, including hemoglobin (HGB), platelet count (PLT), white blood cell (WBC) count, hematocrit (HCT), red cell distribution width (RDW), activated partial thromboplastin time (APTT), prothrombin time (PT), and glucose, were likewise measured within 24 hours before or after invasive mechanical ventilation. Treatment during hospitalization included invasive medical procedures, medication, and blood product transfusions. Invasive medical procedures were defined as surgical operation, arterial catheterization, and central venous catheterization (CVC). Medication included glucocorticoid, chemotherapy, vasopressor, prophylactic anticoagulation, antiplatelet and statin. For patients with in-hospital VTE, data regarding treatment were obtained before the day of VTE diagnosis.

Sample size

It is generally accepted that no more than one independent variable should be included in the model for every 10 events to avoid over-fitting the model [9]. There were 131 patients diagnosed with in-hospital VTE in the training dataset, suggesting that up to 13 variables (131 divided by 10) could safely be considered.

Missing data

Variables with missing data are common in the MIMIC-III database. To avoid bias due to the high volume of missing data, variables with over 30% missing values were not included in this study, such as body mass index (BMI). Missing values from variables with less than 30% missing values were imputed using random forest.

Statistical analysis

All continuous variables were non-normally distributed, which was confirmed using the Shapiro-Wilk test. Accordingly, continuous variables were expressed as the median (interquartile ranges, IQRs) and categorical variables as numbers (percentages). To reduce the effect of outliers and increase convenience in clinical use, continuous variables were categorized into discrete groups. In Risk Assessment Model I, receiver operating characteristic (ROC) analyses were conducted to identify the optimal cut-off point. The continuous variables were converted into binary variables, which may lead to loss of data. Therefore, we attempted to convert continuous variables into multi-categorical variables in Risk Assessment Model II for model updating and conditional inference trees (as implemented in the smbinning procedure in R) analyses were conducted to identify the optimal cut-off point. Subsequently, in-hospital immobilization and glucose were converted into ordered multi-categorical variables, while others were converted into binary variables. However, the difference between categories could not be measured accurately in ordered multi-categorical variables. Analyzing these according to the coded value imposes that the different categories are equidistant, which may lead to greater error [10]. Therefore, ordered multi-categorical variables were converted into dumpy variables in the follow-up analysis. Two predictive models were constructed in the training dataset during model development. In Risk Assessment Model I, we used least absolute shrinkage and selection operator (LASSO) regression with five-fold cross-validation to remove low information variables. The remaining variables, following the LASSO regression, were included in a multivariable logistic regression analysis to obtain the final model. In Risk Assessment Model II, we conducted multivariable logistic regression using the significant variables identified by stepwise backward-selection multiple logistic regression, with a threshold for candidate elimination of >0.05. The variance inflation factors (VIF) were used to detect multicollinearity of all independent variables. The performance of the new prediction models was evaluated and compared with the ICU-Venous Thromboembolism Score in three datasets using the area under the receiver operakting characteristic curve (AUC), and the goodness of fit was measured by calibration curves. Moreover, 95% bias-corrected bootstrap confidence intervals (CI) were calculated for the C-index, and AUCs were compared with the DeLong test. Besides, decision curve analyses (DCA) were plotted to evaluate the usefulness and applicability of the models with the best diagnostic value in a clinical setting. Finally, we plotted a nomogram for the best model. All analyses above were performed using R software (version 4.0.3, CRAN), and p-values of <0.05 were considered statistically significant.

## Results

Participants and baseline characteristics

A total of 6,734 invasively ventilated patients were included in the MIMIC-III database, of whom 181 (2.7%) patients were diagnosed with VTE after invasive mechanical ventilation, 84 (1.2%) patients had isolated DVT, 40 (0.6%) patients had isolated PE, 38 (0.6%) patients had isolated superficial vein thrombosis, six (0.09%) patients had both DVT and PE, and 13 (0.2%) patients had both superficial vein thrombosis and DVT. The flow diagram of patient selection is shown in Supplemental Figure 6. The training and internal validation datasets consisted of 4,714 and 2,020 invasively ventilated patients, respectively. The number of patients with a diagnosis of VTE after invasive mechanical ventilation included in Nanfang Hospital between February 2016 and August 2018 who were included in the external dataset was 78. In addition, 90 patients without VTE after invasive mechanical ventilation were randomly selected as the control group. In the external dataset, 66 (39.3%) patients had isolated DVT, five (3%) patients had isolated PE, three (1.8%) patients had both DVT and PE, and four (2.4%) patients had both superficial vein thrombosis and DVT. The flow diagram of patient selection is presented in Supplementary Figure 7. The baseline characteristics of the three datasets are shown in Table 1.

**Table 1:** Baseline characteristics of patients. APTT: activated partial thromboplastin time; HCT: hematocrit; HGB: hemoglobin; PLT: platelet; PT: prothrombin time; RDW: red cell distribution width; VTE: venous thromboembolism; WBC: white blood cell

Characteristics	Training dataset (N = 4,714)	Internal validation dataset (N = 2,020)	External validation dataset (N = 168)
VTE, n (%)	131 (2.8)	50 (2.5)	78 (46.4)
Demographics			
Age, year (median [IQR])	65.00 [54.00, 76.00]	66.00 [54.00, 77.00]	59.00 [46.00, 72.00]
Gender, male, n (%)	2,872 (60.9)	1,229 (60.8)	114 (67.9)
Prior history of VTE, n (%)	178 (3.8)	81 (4.0)	2 (1.2)
In-hospital immobilization, d (median [IQR])	3.00 [1.00, 7.00]	3.00 [1.00, 7.00]	7.00 [3.00, 13.00]
Symptoms			
Edema of extremities, n (%)	2,287 (48.5)	977 (48.4)	45 (26.8)
Pain in extremities or chest pain, n (%)	1,134 (24.1)	491 (24.3)	36 (21.4)
Vital signs			
Heart rate, beats per minute (median [IQR])	103.00 [92.00, 116.00]	103.00 [91.00, 117.00]	91.00 [80.00, 108.25]
Systolic blood pressure, mmHg (median [IQR])	148.00 [135.00, 163.00]	146.00 [134.00, 163.00]	124.00 [112.00, 138.00]
Diastolic blood pressure, mmHg (median [IQR])	80.00 [71.00, 92.00]	79.00 [71.00, 91.00]	70.50 [64.00, 80.25]
SpO_2_, % (median [IQR])	94.00 [91.00, 96.00]	94.00 [91.00, 96.00]	95.00 [89.49, 98.00]
Respiratory rate, beats per minute (median [IQR])	26.00 [23.00, 30.00]	26.00 [23.00, 30.00]	24.00 [20.00, 30.00]
Comorbidities			
Chronic heart failure, n (%)	983 (20.9)	443 (21.9)	11 (6.5)
Chronic lung disease, n (%)	1,157 (24.5)	501 (24.8)	26 (15.5)
Diabetes mellitus, n (%)	1,341 (28.4)	581 (28.8)	42 (25.0)
Hypertension, n (%)	2,912 (61.8)	1217 (60.2)	73 (43.5)
Liver disease, n (%)	402 (8.5)	181 (9.0)	26 (15.5)
Malignant tumor, n (%)	1,084 (23.0)	478 (23.7)	36 (21.4)
Infection, n (%)	2,031 (43.1)	865 (42.8)	113 (67.3)
Acute myocardial infarction, n (%)	455 (9.7)	188 (9.3)	18 (10.7)
Atrial fibrillation or atrial flutter, n (%)	1,474 (31.3)	619 (30.6)	37 (22.0)
Stroke, n (%)	243 (5.2)	97 (4.8)	35 (20.8)
Trauma, n (%)	654 (13.9)	288 (14.3)	8 (4.8)
Treatment			
Invasive medical procedures			
Surgical operation, n (%)	2,871 (60.9)	1,204 (59.6)	101 (60.1)
Arterial catheterization, n (%)	3,192 (67.7)	1,350 (66.8)	111 (66.1)
Central venous catheterization, n (%)	2,524 (53.5)	1 060 (52.5)	147 (87.5)
Medication			
Glucocorticoid, n (%)	979 (20.8)	420 (20.8)	92 (54.8)
Chemotherapy, n (%)	60 (1.3)	15 (0.7)	5 (3.0)
Vasopressor, n (%)	2,823 (59.9)	1,226 (60.7)	108 (64.3)
Prophylactic anticoagulation, n (%)	2,504 (53.1)	1,045 (51.7)	71 (42.3)
Antiplatelet, n (%)	2,689 (57.0)	1,194 (59.1)	50 (29.8)
Statin, n (%)	2,227 (47.2)	975 (48.3)	39 (23.2)
Blood product transfusions			
Red blood cell transfusion, n (%)	1,796 (38.1)	768 (38.0)	34 (20.2)
Platelet transfusion, n (%)	562 (11.9)	256 (12.7)	16 (9.5)
Fresh frozen plasma transfusion, n (%)	662 (14.0)	292 (14.5)	39 (23.2)
Laboratory variables			
HGB, g/dL (median [IQR])	10.90 [9.50, 12.40]	10.80 [9.60, 12.40]	10.95 [8.80, 12.30]
PLT, 10^3^/μL (median [IQR])	193.00 [140.00, 256.75]	191.00 [140.00, 262.00]	154.00 [103.00, 206.50]
WBC, 10^3^/μL (median [IQR])	11.90 [8.60, 15.90]	12.00 [8.80, 16.10]	11.76 [9.16, 15.33]
HCT, % (median [IQR])	32.20 [28.30, 37.00]	32.10 [28.50, 37.00]	33 [27.00, 37.00]
PT, seconds (median [IQR])	14.40 [13.10, 16.10]	14.30 [13.10, 16.00]	13.75 [12.50, 15.70]
APTT, seconds (median [IQR])	31.00 [26.70, 38.00]	30.90 [26.70, 38.02]	32.80 [26.90, 40.15]
RDW, % (median [IQR])	14.20 [13.40, 15.40]	14.20 [13.40, 15.40]	14.20 [13.20, 16.52]
Glucose, mg/dL (median [IQR])	126.00 [106.00, 157.00]	125.00 [104.00, 156.00]	130.77 [102.69, 173.52]

Risk Assessment Model development

**Figure 1:**
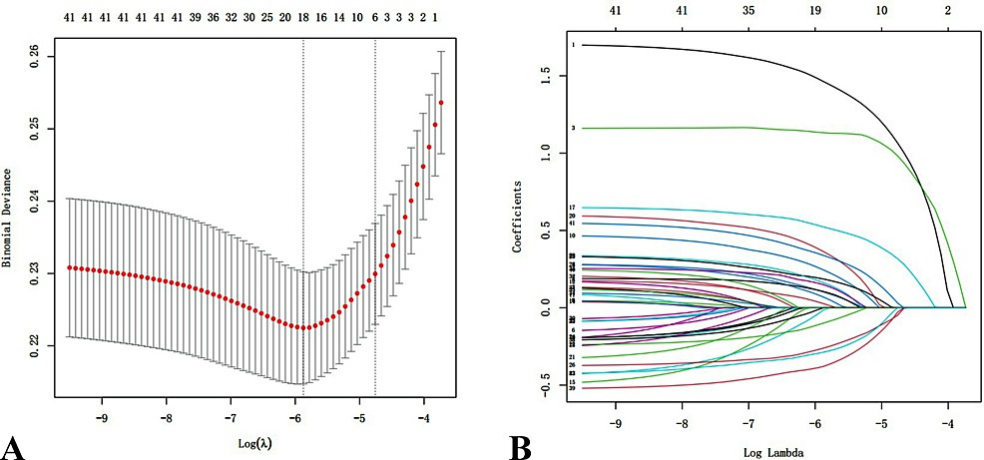
(A) Tuning parameter (lambda) was selected in the LASSO analysis by five-fold cross-validation. With the lambda value of 0.0086, six characteristics were included. (B) The coefficients of variables in LASSO analysis. LASSO: least absolute shrinkage and selection operator

**Figure 2:**
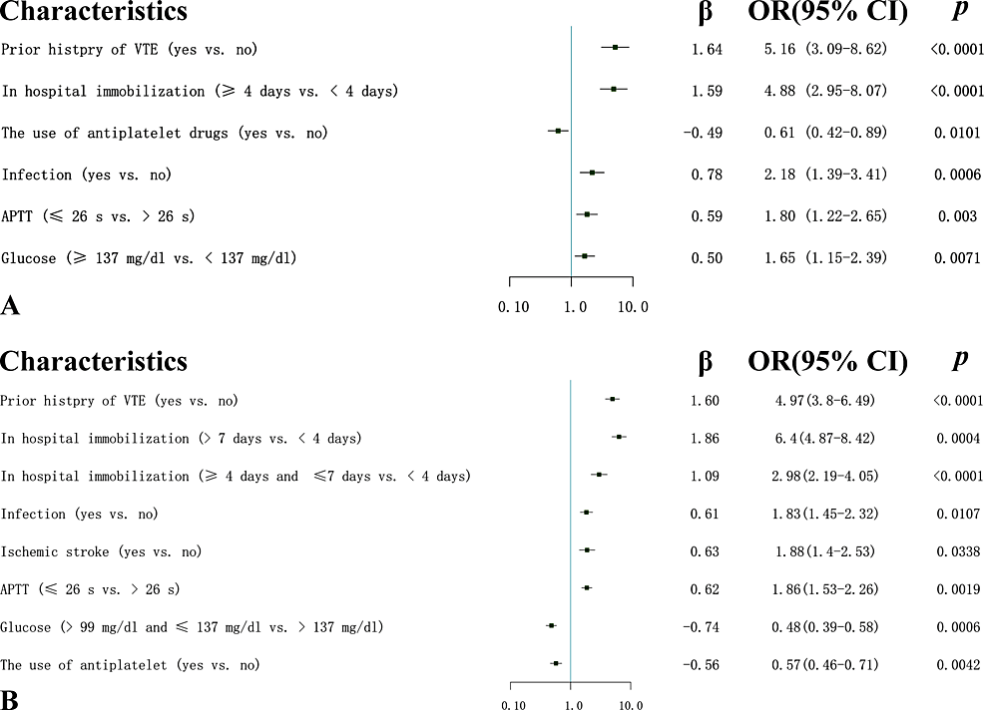
(A) Six predictive factors were included in the Risk Assessment Model I. (B) Seven predictive factors were included in the Risk Assessment Model II. APTT: activated partial thromboplastin time; VTE: venous thromboembolism; OR: odds ratio; CI: confidence interval

In Risk Assessment Model I, 43 characteristics were narrowed down to six potential predictors by LASSO regression analysis in the training dataset (Figure 1). Multivariate analysis showed that glucose level of greater than or equal to 137 mg/dL, infection, APTT of less than or equal to 26 seconds, and in-hospital immobilization of longer than or equal to four days were independent risk factors for VTE, while the use of antiplatelet treatment was an independent protective factor for VTE (Figure 2A). All variables had a VIF of less than 10, indicating the absence of multicollinearity. In Risk Assessment Model II, the stepwise backward-selection multiple logistic regression was applied to select factors that had the highest association with VTE in the training dataset (p < 0.05). The results showed that seven predictive factors, namely, prior history of VTE, in-hospital immobilization, infection, ischemic stroke, glucose, the use of antiplatelet, and APTT, were selected. We then built a prediction model using binary logistic regression analysis based on these seven features (Figure 2B): prior history of VTE (OR = 4.97, 95% CI = 3.8-6.49, p < 0.0001), in-hospital immobilization (≥4 days and ≤7 days vs. <4 days, OR = 2.98, 95% CI = 2.19-4.05, p < 0.0001), in-hospital immobilization (>7 days vs. <4 days, OR = 6.4, 95% CI = 4.87-8.42, p = 0.0004), infection (OR = 1.83, 95% CI = 1.45-2.32, p = 0.0107), ischemic stroke (yes vs. no, OR = 1.88, 95% CI = 1.4-2.53, p = 0.0338), APTT (≤26 seconds vs. >26 seconds, OR = 1.86, 95% CI = 1.53-2.26, p = 0.0019), glucose (>99 mg/dL and ≤ 137 mg/dL vs. > 137 mg/dL, OR = 0.48, 95% CI = 0.39-0.58, p = 0.0006), and the use of antiplatelet (yes vs. no, OR = 0.57, 95% CI = 0.46-0.71, p = 0.0042). All variables had a VIF of less than two, indicating the absence of multicollinearity.

Risk Assessment Model performance and comparison

**Figure 3:**
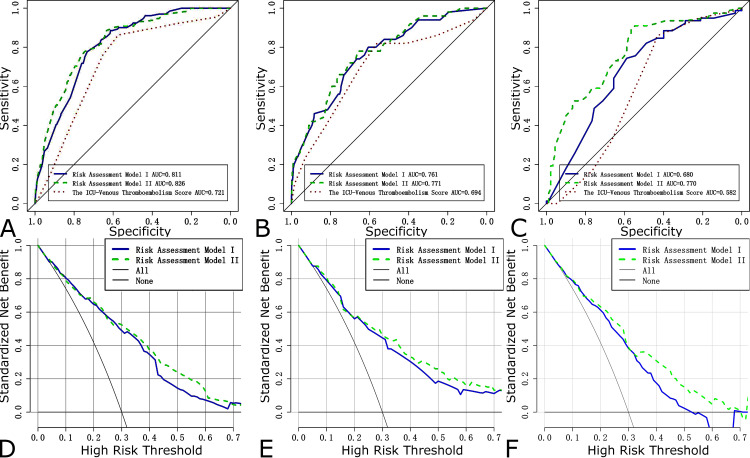
ROCs for the training dataset (A), internal validation dataset (B), and external dataset (C). DCAs for the training dataset (D), internal validation dataset, (E) and external dataset (F). ROC: receiver operating characteristic curve; DCA: decision curve analysis

**Figure 4:**
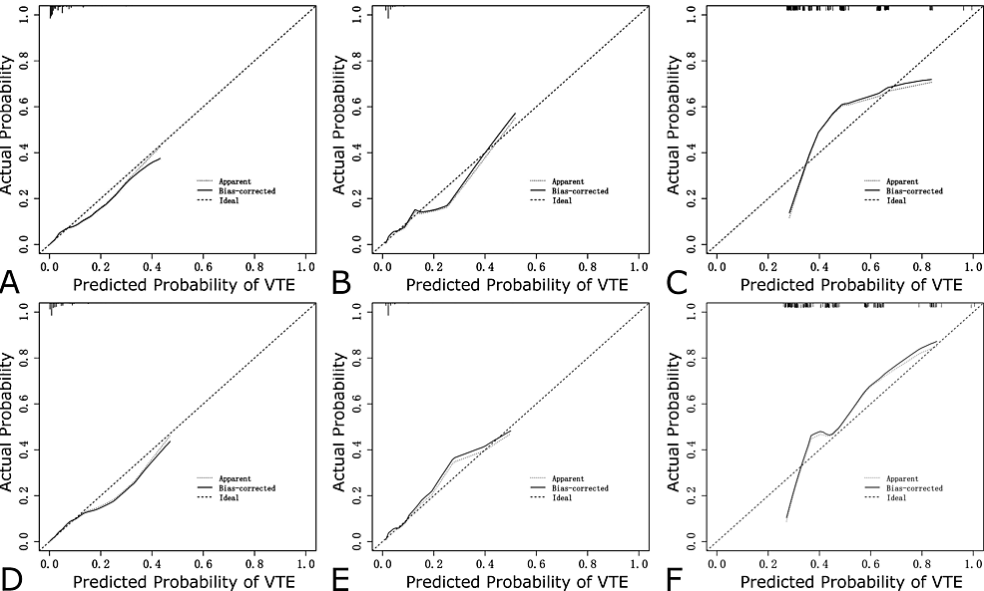
Calibration curves for Risk Assessment Model I (A for the training dataset, B for the internal validation dataset, and C for the external validation dataset) and Risk Assessment Model II (D for the training dataset, E for the internal validation dataset, and F for the external validation dataset). VTE: venous thromboembolism

**Figure 5:**
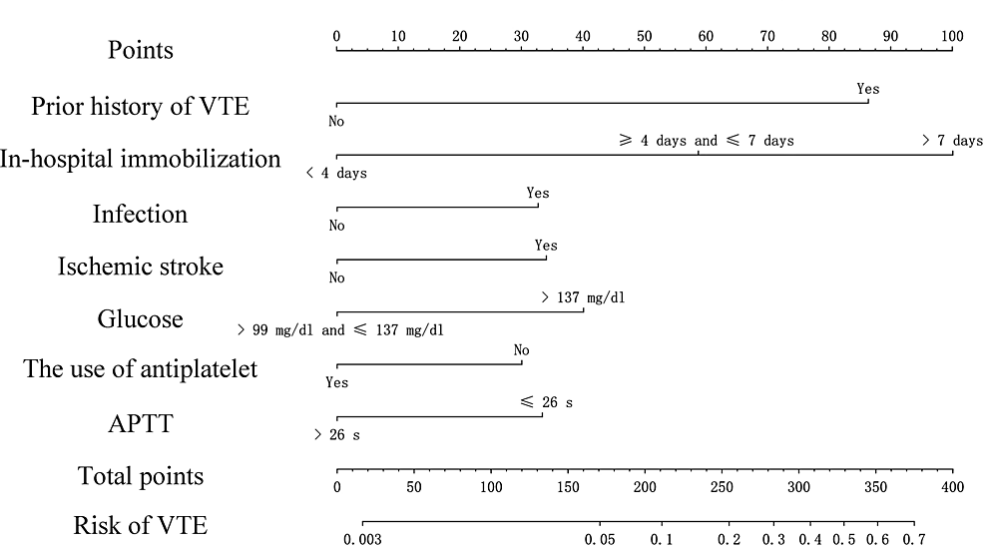
Nomogram of VTE absolute risk prediction in invasively ventilated patients (Risk Assessment Model II). APTT: activated partial thromboplastin time; VTE: venous thromboembolism

AUCs for Risk Assessment Model I, Risk Assessment Model II, and the ICU-Venous thromboembolism Score were 0.826 (95% CI = 0.793-0.858), 0.811 (95% CI = 0.780-0.841) and 0.721 (95% CI = 0.681-0.761) in the training dataset (Figure 3A), respectively, showing good discriminatory power. DeLong’s test revealed that the test AUC of Risk Assessment Model II was significantly larger than Risk Assessment Model I (p = 0.048) and the ICU-Venous Thromboembolism Score (p < 0.001). In the internal validation dataset, threes models had AUCs of 0.761 (95% CI = 0.694-0.828), 0.771 (95% CI = 0.706-0.836) and 0.694 (95% CI = 0.615-0.774), respectively (Figure 3B). DeLong’s test revealed that there were no statistically significant differences between them in the internal validation dataset. The AUCs for Risk Assessment Model I, Risk Assessment Model II, and the ICU-Venous thromboembolism Score were 0.680 (95% CI = 0.599-0.761), 0.770 (95% CI = 0.699-0.841) and 0.582 (95% CI = 0.496-0.668) in the external validation dataset (Figure 3C), respectively. DeLong’s test suggested that Risk Assessment Model II was a better discriminator of VTE in invasively mechanical ventilation patients than Risk Assessment Model II (p = 0.0046) and the ICU-Venous thromboembolism Score (p < 0.001) in the external validation dataset. According to the results of DCA, the net benefit of Risk Assessment Model II was larger over the range of Risk Assessment Model I in the training dataset (Figure 3D), internal validation dataset (Figure 3E), and external validation dataset (Figure 3F), which means Model II is optimal. The calibration curves demonstrated a satisfying agreement for the Risk Assessment Model I and Risk Assessment Model II in all datasets (Figure 4). To sum up, Risk Assessment Model II performed best, which indicated Model II possesses significant predictive value. The nomogram of VTE absolute risk prediction was established in invasively ventilated patients with the prediction factors obtained from Risk Assessment Model II (Figure 5).

 

## Discussion

Patients with invasive mechanical ventilation may be at high risk of acquiring VTE. Venous stasis results from muscular paralysis, high positive end-expiratory pressure, and injuries or occlusions of the pulmonary microvascular network in invasively mechanical ventilated patients [11]. Besides, the clinical manifestations of VTE are usually atypical in this condition (invasive mechanical ventilation) due to disturbance of consciousness, the requirement of sedation, or underlying medical illnesses such as congestive heart failure [12]. Therefore, establishing a diagnosis of VTE in invasively mechanical ventilated patients is more likely to be delayed or missed [13], which indicates the urgent need to develop reliable assessment models to identify patients with invasive mechanical ventilation who are at an increased risk of suffering from VTE.

In this study, two models were promoted in different ways aiming to predict the risk of VTE in patients with invasive mechanical ventilation. In Risk Assessment Model I, the continuous variables were converted into binary variables, which may lead to loss of data. Therefore, we attempted to convert continuous variables into multi-categorical variables in Risk Assessment Model II for model updating. The Risk Assessment Model II was superior in predicting VTE in invasively mechanical ventilated patients compared with Risk Assessment Model I and the ICU-Venous Thromboembolism Score.

A strong association between VTE and in-hospital immobilization was detected in the model, which was captured according to electronic medical records charted at the bedside of the patient. Due to loss of consciousness, patients with invasive mechanical ventilation usually experience no muscle contraction and muscle tension, causing serious impairment to venous reflux. With the prolongation of immobilization, the blood stasis is more serious. The reported duration of immobilization beyond which VTE risk increases differs among studies. In a retrospective study that included 2,188 consecutive neurological ICU patients, VTE was associated with a longer duration of immobilization (OR = 1.07 per day, 95% CI = 1.05-1.09) [14]. Immobilization for more than or equal to seven days was an independent risk factor associated with three-month VTE (HR = 1.9, 95% CI = 1.3-2.7) in a study of 15,125 hospitalized medical patients [15]. In this study, we found that patients with a duration of in-hospital immobilization ranging between four and seven days had an OR for VTE of 2.98 (95% CI = 2.19-4.05) while patients with a duration of more than seven days had an OR for VTE of 6.4 (95% CI = 4.87-8.42) compared with patients immobilizing for less than four days.

Infections have been reported to increase the risk of VTE by 2-20 times and were almost associated with double the risk of VTE in our study. The highest risk of VTE is when the infection is active or within a few weeks after the infection [16]. The key point that underpins the risk of VTE is the level of inflammation induced by infection, which can lead to a procoagulant state. The release of inflammatory factors activates platelets, which may be accompanied by endothelial damage, leading to fibrin deposition and thrombosis [17].

Long-term immobilization, age, and infection are well-known risk factors for VTE, which are common in patients with ischemic stroke. The incidence of DVT within two weeks after acute ischemic stroke ranges from 27% to 75% [18]. In a prospective multi-center study, among 1,380 cases, 4.49% (62 cases) had DVT and 0.80% (11 cases) had PE following acute stroke [19]. Among 30,002 participants recruited from three surveys of the Tromsø study (conducted in 1994-1995, 2001, and 2007-2008), 1,360 participants suffered from ischemic stroke and 722 developed a VTE, and ischemic stroke was associated with an increased risk of VTE (HR = 3.2, 95% CI = 2.4-4.4) [20]. For patients with invasive mechanical ventilation, ischemic stroke may result in prolonged mechanical ventilation and immobilization, which may aggravate blood stasis.

A shortened APTT is believed to represent a procoagulant tendency, which can occur in patients with malignant tumors, disseminated intravascular coagulation, etc. In a retrospective study including 13,880 patients with a median follow-up period of 13.1 years, compared with participants in the fourth quartile of APTT, participants in the lowest two quartiles of APTT had a 2.4-fold (95% CI = 1.4-4.2) and a 1.9-fold (95% CI = 1.1-3.2) higher risk of VTE, respectively [21]. In a case-control study, patients with an APTT ratio lower than the fifth percentile of the distribution in the control group had an OR for VTE of 2.4 (95% CI = 1.7-3.6) [22].

Moderate glucose control within 24 hours before or after invasive mechanical ventilation was confirmed in this study to decrease the risk of VTE. Studies on the effect of glucose levels on VTE are scarce and have reported conflicting results. A case-control study found that blood glucose levels of fasting patients did not increase the risk of VTE (OR = 0.98, 95% CI = 0.69-1.37) [23]. However, another case-control study indicated that increased risk of VTE was related to hyperglycemia (OR = 2.21, 95% CI = 1.2-4.05) [24]. Stegenga et al. [25] showed that experimentally induced acute hyperglycemia has a strong procoagulant effect in healthy volunteers. Hyperglycemia activated coagulation through endothelial glycocalyx damage, upregulation of tissue factors, non-enzymatic glycation, and the increase of oxidative stress [26].

The use of antiplatelet was identified as a protective factor for VTE in invasively ventilated patients. Platelets have been shown to be involved in the process of VTE. Activated platelets promote VTE by releasing polyphosphates, inflammatory factors, phosphatidylserine, and particles that expose tissue factors while stimulating the formation of an extracellular bactericidal network of neutrophils that provide the backbone for platelet adhesion [27]. Although antiplatelet agents are not recommended alternatives to prophylactic anticoagulation in VTE patients, aspirin may play a role in the prevention of recurrent VTE among patients who have decided to stop receiving anticoagulants [28]. Moreover, the orthopedic surgery community has long embraced aspirin as an effective option in VTE prevention for high-risk orthopedic surgery patients [29]. At present, no clinical trials exist that relate the use of antiplatelet agents in critically ill patients in prophylactic anticoagulation. In a retrospective study of 193 mechanically ventilated patients, multivariate regression analysis showed that the use of aspirin during hospitalization could reduce the risk of DVT (OR = 0.39, 95% CI = 0.16-0.94) [30].

This study has several advantages. First, to our knowledge, this is the first study in which risk assessment models for VTE in patients with invasive mechanical ventilation are developed. Second, this study was conducted under the recommendations of the TRIPOD statement. Third, the statistical issues surrounding the development and validation of models merit discussion. In the training dataset, we confirmed 131 patients developing VTE and our final model contained seven variables, consistent with the sample size estimation. Statistical methods were also used to handle missing data. We performed statistical analyses to isolate the risk factors related to the VTE and avoided over-fitting. Fourth, two models were both validated in an independent dataset of patients from the same database and another independent dataset from Nanfang Hospital.

As the results showed, the new prediction model performed better than the ICU-Venous Thromboembolism Score. Even though the ICU-Venous Thromboembolism Score is mainly used to assess the risk of DVT and PE in critically ill patients, we included patients with superficial vein thrombosis in our study. Besides, our study focused on patients with invasive mechanical ventilation, while mechanical ventilation is one of the predictive factors in the ICU-Venous Thromboembolism Score. Therefore, prospective validation in a larger population is necessary to further evaluate the performance of the score.

One limitation of our study is that it is a retrospective study, and data on non-pharmacological prophylactic intervention, hereditary risk factors for thrombosis, BMI, and D-dimer were not used due to the high volume of missing data. Moreover, the incidence of VTE was lower than anticipated, which may lead to model over-fitting and wide confidence intervals. Nevertheless, the results of calibration curves suggested good agreement between the predicted and observed values for both models in the external validation dataset. Additionally, data on time-dependent prospective factors were not collected during invasive mechanical ventilation.

## Conclusions

Of the two simple risk assessment models designed in this study, Risk Assessment Model II has a significantly improved performance compared with the currently available assessment tool, namely, the ICU-Venous Thromboembolism Score, which was based on seven clinical and laboratory parameters for VTE in invasively ventilated patients. The model can detect the high risk of VTE population in patients with invasive mechanical ventilation, reducing its burden.

## Figures and Tables

**Figure 6:**
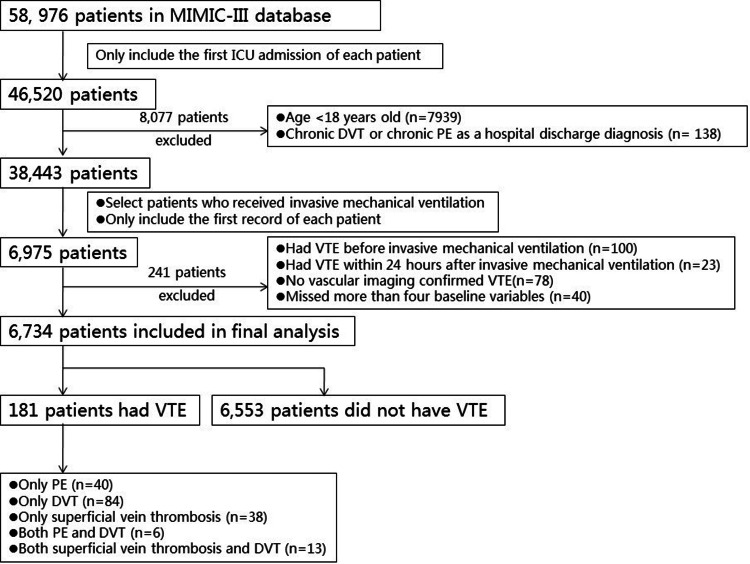
Study population (the training group and internal validation group). DVT: deep vein thrombosis; PE: pulmonary embolism; VTE: venous thromboembolism

**Figure 7:**
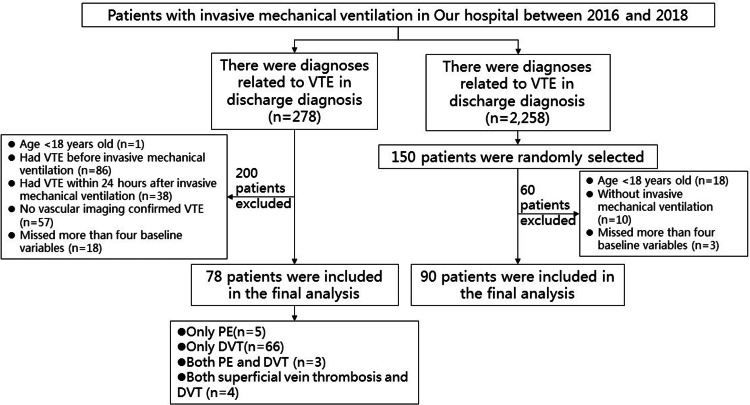
Study population (the external validation group). DVT: deep vein thrombosis; PE: pulmonary embolism; VTE: venous thromboembolism
